# Parenting support in ECEC services: the views of practitioners implementing a model in the Irish context about parents’ engagement and associated outcomes

**DOI:** 10.3389/fsoc.2025.1489477

**Published:** 2025-04-25

**Authors:** Catarina Leitão, Jefrey Shumba

**Affiliations:** Childhood Development Initiative, Dublin, Ireland

**Keywords:** caregivers, parents, parenting support, early childhood education and care, preschool, kindergarten, intervention, practitioners

## Abstract

**Introduction:**

Providing parenting support in combination with Early Childhood Education and Care (ECEC) can positively impact children’s and families’ wellbeing. This study aimed to explore factors potentially affecting parents’ engagement in a parenting support model in ECEC services and associated outcomes. This model involves a professional role dedicated to working with parents, the Parent Carer Facilitator (PCF).

**Methods:**

Eight PCFs and seven managers of ECEC services in Ireland implementing this model were interviewed. Data were analysed through thematic analysis.

**Results:**

Four themes were generated: factors related to parents’ engagement, approaches to promoting engagement and responsiveness to families’ needs, implementation drivers and relevance of the model.

**Discussion:**

Findings indicated that a professional role dedicated to supporting parents within ECEC services, focusing on establishing trusting relationships and tailoring support according to families’ needs, can foster parents’ engagement in the support offered and positive outcomes regarding parenting and parents-ECEC service relationship.

## 1 Introduction

Supporting children and their families through quality services is a rights matter (Council Recommendation, 2021). European policy orientations have increasingly focused on supporting caregivers in their parenting to positively impact children’s development and family wellbeing (Devaney and Crosse, 2023; Dolan et al., 2020). Providing information and services for parents (herein referring to caregivers in a parenting role) to strengthen their knowledge, confidence and skills in child-rearing and wellbeing can be described as parenting support (Daly, 2022; WHO, 2022). Combining parenting support with early childhood education and care (ECEC) services can enhance children’s cognitive and socio-emotional outcomes and parents’ awareness about child development (Koshyk et al., 2020; Luo et al., 2022; Sheridan et al., 2019; Smith et al., 2020). However, published research on parenting support in Europe often describes programmes in social and health services delivered over a set number of sessions in a group format (Bernedo et al., 2024).

Given the ongoing interactions between parents and ECEC services, the latter have the potential to increase access to parenting support, a recognised need in Europe, including Ireland (DCEDIY, 2022, 2024; OECD, 2021), the context of the current study. Nationally, state provision of parenting supports has occurred directly and through commissioned services at the local level depending on need, particularly in areas of disadvantage. Still, these supports have not been consistently promoted through the ECEC sector (DCEDIY, 2024).

The current study explored the views of practitioners delivering a parenting support model, Powerful Parenting, which involves an ongoing professional Parent Carer Facilitator (PCF) role dedicated to supporting parents in ECEC services.

### 1.1 Powerful parenting: a parenting support model in ECEC services

A non-governmental organisation in Ireland, the Childhood Development Initiative, developed Powerful Parenting and has coordinated it in consultation with the community ECEC services implementing it (eight in the same town at the time of this study), with state funding support. It involves one PCF per ECEC service, 25 h a week, whose support is available to all parents with children attending the ECEC service or referred by other services. Powerful Parenting is based on a previous support approach whose evaluation indicated that intervention group parents reported receiving extra and more types of help from their ECEC service than their comparison counterparts (Hayes et al., 2013).

PCFs’ responsibilities encompass assisting parents in identifying their children’s or own needs and offering support to promote their wellbeing, including by enhancing parents’ confidence and competence in parenting. The identified needs inform the implemented supports/activities, which can vary across ECEC services (for this reason, Powerful Parenting is described as a model rather than a standardised programme). The model follows a strengths-based approach (building on parents’/families’ strengths rather than focusing on fixing deficits) and positive parenting principles (emphasising nurturing, encouragement, and guidance), acknowledged as relevant to support parents and families effectively (Devaney et al., 2021; Dunst, 2023; WHO, 2022).

The PCF can support parents through one-to-one and group work in the ECEC setting, remotely or at the families’ home. The support can be informational (e.g., information on children’s development), emotional (e.g., listening to parents’ concerns and promoting the mobilisation of resources), and practical (e.g., assisting in accessing services; Supplementary Table ST1 shows the number of referrals to other services reported by PCFs during the academic year). PCFs can implement the Parents Plus Early Years Programme (e.g., Sharry et al., 2003) through up to 12 sessions, whose studies in Ireland indicated improvements in children’s behavioural outcomes, parental stress, and the home learning environment (Gerber et al., 2016; Griffin et al., 2010; Hayes et al., 2013). PCFs can also organise activities with other ECEC practitioners for parents and children.

The PCF role requires a third-level degree in Childcare, Social Work/Care, Psychology or equivalent, and a minimum of 3 years experience working with parents. The organisation coordinating the model provides PCFs with training on Parents Plus, quality implementation, restorative practices, and monitoring/evaluation. The same organisation holds monthly community of practice (CoP) meetings with the group of PCFs and two annual planning meetings with PCFs and managers of the ECEC services implementing the model. These meetings provide opportunities for practitioners to reflect on their work with families, which can support continuous professional development (Slot and Nata, 2019).

### 1.2 The current study

In published literature on parenting support in Europe, there seems to be less emphasis on or acknowledgement of the need to support families over long periods or the potential value of ‘real-time’ support (Devaney et al., 2021; Dolan et al., 2020). Researched-supported parenting support programmes implemented in ECEC services in Europe and Ireland often encompass a defined number of modules or sessions offered in a group format (Bernedo et al., 2024; Leitão, 2023). Examples include the Parents Plus Early Years Programme (e.g., Sharry et al., 2003),^1^ Peep the Learning Together Programme,^2^ and the Incredible Years Parenting Programme (Webster-Stratton, 2001).^3^ Powerful Parenting can be considered an innovative model in parenting support as it involves an ongoing available professional role in ECEC services, the PCF, offering tailored support to respond to parents’/families’ needs, individually or in group, and at the service or the family’s home.

Powerful Parenting also includes characteristics identified as promoting parents’ engagement in parenting support interventions and positive outcomes, such as: accessible and tailored support according to parents’/families’ needs, a focus on more than one area of need, and coordination with other services for children and families (Anders et al., 2019; Cadima et al., 2017; Molinuevo, 2013; Moran et al., 2004). Still, further research on factors affecting engagement and outcomes regarding parenting support in ECEC services has been recognised as necessary (Britto et al., 2022; Cook et al., 2023; Sim et al., 2021). Studying an innovative model, such as Powerful Parenting, can contribute to informing about these factors.

Practitioners working directly with parents/families can majorly affect the outreach and effectiveness of family and parenting supports (Canavan et al., 2016; Cohen et al., 2020). However, these practitioners are still not often represented in family support networks in European countries (Jiménez et al., 2024), highlighting the need to consider their views in research aiming to inform policy and practice development.

This study’s research question was about what factors can affect parents’ engagement in a model such as Powerful Parenting and associated outcomes. It was addressed by collecting PCFs’ and ECEC managers’ views on Powerful Parenting organisation, utilisation, quality, and perceived outcomes.

## 2 Materials and methods

### 2.1 Participants

Eight PCFs and seven managers (one coordinated two ECEC services) from the eight ECEC services delivering Powerful Parenting participated in this study. All PCFs were females; seven PCFs spoke English as their first language; on average, they had almost 6 years of experience in the PCF role (M = 5.65; SD = 7.19), ranging between 9 months and 21 years. Six ECEC managers were female, and one was male; all spoke English as their first language; on average, they had almost 17 years of experience as ECEC managers (M = 16.50; SD = 3.21), ranging between 13 and 20 years.

All ECEC services were located in an area identified as economically disadvantaged (Central Statistics Office, 2022; Haase and Pratschke, 2017). At the time of the study, 213 children between three and 6 years old attended the eight services, varying from 10 to 68 children per service (M = 26.63; SD = 20.28). The child-to-staff ratio varied between four and eight children to one staff member.

### 2.2 Instruments

Table 1 shows the semi-structured interview protocols used. Some questions differed between PCFs and managers, given their different roles within Powerful Parenting (PCFs directly worked with parents and managers with PCFs). The study’s first author developed the protocols by consulting the organisation coordinating the model and previous literature (e.g., Barata et al., 2016; Hayes et al., 2013).

**Table 1:** Interview protocol.

Dimensions	Questions	Participants
Organisation	Can you share examples of activities organised within your service during this academic year?	PCFs Managers
	Have the activities been implemented as planned or intended?	
	How have the activities been planned?	
	What supports or resources have been available to support activities in the service? What other supports would be useful?	
Utilisation	How do you perceive parents’ interest in using the model?	
	What can constitute barriers to parents’ participation?	
	What can enable parents’ participation?	PCFs
	What have been the interests and needs of the parents/families that use the model?	
Quality	How do you address the needs of the parents/carers?	
	What are the most positive aspects of this model?	PCFs Managers
	What are the less positive aspects of this model? Do you have suggestions for improvement?	
Perceived outcomes	In your view, from 1 (low) to 5 (high), to what extent is the model addressing or strengthening the following? Strengthen parenting skills Help parents/carers to understand the child’s development Provide parents/carers with new ways to interact with children Promote the home learning environment Promote improved partnerships between the parents/carers and the ECEC services Promote the engagement of both parents of the child or other carers in children’s education Help families prepare for a smoother transition of children to school Involve families with other community services. Is there any other benefit that you think the model is having?	PCFs
	What are the main benefits for families?	Managers
Other	In general, how do you think the model is going?	PCFs Managers
	Is there anything else you’d like to say?	

### 2.3 Data collection

The first author invited and interviewed the participants individually via telephone or online conference tool (May–June 2021). Thirteen interviews were audio-recorded with the consent of the participants. Notes were taken instead during two interviews (following PCF 1’s and Manager 3’s preference).

### 2.4 Data analysis

Qualitative data were analysed through thematic analysis following Braun and Clarke (2006). Both authors independently read the interview transcripts. The second author coded the data by adding notes to the text, which were then independently reviewed by the first author. Inconsistencies were discussed between the authors until a consensus was reached. This process was first conducted with PCFs’ data and then with managers’ data. Notes generated from PCFs’ and managers’ data were compared, and similar ones were collated. A set of codes was developed based on the notes (including the ones collated and those kept separated). While revisiting the data, the authors collaboratively pulled similar codes to form sub-themes and themes. Both authors agreed on the selected quotes for the study’s write-up, aiming to include the views of different participants. The Results section includes references indicating the practitioners (PCFs and/or managers) on who the findings were based.

Descriptive statistics are presented for quantitative data. When PCFs indicated two scores (four or five) regarding the same perceived outcome and were not requested for a specific score (e.g., to avoid breaking the interview flow), the response was treated as missing data.

Preliminary results were shared with the participants for validation.

## 3 Results

Four themes and 13 sub-themes were generated.

### 3.1 Theme 1. Factors related to parents’ engagement

Based on PCFs’ and managers’ views, there seemed to be an overall acknowledgement of high parental engagement with Powerful Parenting. *‘I think the majority, the 99.9% of the parents, are definitely willing to participate’* (PCF 3). However, the engagement was affected by the COVID-19 pandemic and could vary depending on the implemented activities and parents’ circumstances.

#### 3.1.1 Impact of COVID-19 on engagement

According to PCFs and managers, during the academic year this study occurred, parents seemed to show a high interest in the proposed activities and *‘a real bond between them’* (PCF 3), which could be related to staying-at-home measures and being more socially isolated. *‘I do not know whether that’s the influence of being stuck at home for so long, and now they are just looking to be involved with everything’* (PCF 8). Particularly, PCFs and managers noticed an increased engagement of fathers. Previously, *‘dads were at work, so it was harder to get them engaged in what was happening’* (Manager 2).

During the same academic year, PCFs organised online activities, offered support via telephone or text, and sent resources to families’ homes (Supplementary Table ST2 shows examples of supports/activities). However, despite concerns about being infected with COVID-19, PCFs and managers noted parents’ preference for face-to-face activities, including due to challenges with technology. ‘*With face-to-face, they seem to engage a little bit more and tell us more about what’s going on in their lives*’ (PCF 8). A manager expressed an exception: *‘There are very young parents who will be very hesitant to engage. The PCF would send them out things* [online]*, and that has worked well’* (Manager 6).

#### 3.1.2 Barriers to parents’ engagement besides the pandemic

According to PCFs and managers, simultaneous commitments could affect parents’ engagement in the activities/supports, such as ‘*their work hours*’ (PCF 6) and *‘having smaller children and not being able to arrange childcare to come in’* (Manager 7).

Other identified barriers by PCFs and managers included lack of interest, which could vary depending on the activity topic, lack of confidence, and language barriers when parents’ first language was not English. ‘*Barriers can include something that might not interest them, or they might have other problems. They might think if they will be judged or not. Lack of confidence can also be a barrier*’ (PCF 1). According to one manager, parents’ lower engagement could also be related to not perceiving themselves as experts in their children’s education. ‘*They kind of hand over the expertise, not realising the expertise they have already carried to the door*’ (Manager 4).

PCFs’ and managers’ reports indicated that difficult life situations could also lead to less capacity or availability from parents to engage, including when perceiving the support within Powerful Parenting as similar to previous services. ‘*A lot of our families are already involved with social workers and child protection services. It just feels like any proposition would be seen as something similar*’ (PCF 2).

#### 3.1.3 Activities/supports with high engagement

PCFs noted one-to-one meetings (e.g., during morning drop-off) as moments parents looked for, which could be related to the opportunity to share more about their lives. ‘*I had a lot of face-to-face meetings individually, and people are really looking for it*’ (PCF 2). Based on PCFs’ and managers’ views, parents also seemed particularly interested in activities involving them and their children or whose rationale was to benefit their children. ‘*When we offered things tailored to the parents, the uptake was there, particularly if their child was involved’* (Manager 4).

### 3.2 Theme 2. Approaches to promote engagement and responsiveness to needs

Identified approaches to promote the engagement of parents and responsiveness to their children or own needs included building a relationship with parents, tailoring support, and adapting activities during the pandemic.

#### 3.2.1 Building a relationship with parents

Based on PCFs’ and managers’ views, building a trusting relationship with parents seemed relevant to promoting their engagement in the support offered. ‘*They have to feel comfortable to be able to open up and share their lives with you. So, it is kind of meeting them where they are at, at their pace, and building your relationship with them*’ (PCF 6).

Building a relationship could involve moving at the parents’ pace and using an empathic/non-judgemental approach, ‘*listening carefully and respectfully, with the awareness that the parents are experts in their own lives and what works for them*’ (PCF 1). It could encompass keeping an open line of communication with parents, such as sharing the rationale of the proposed supports. ‘*I try to be very honest with the parents. I explain all that we are offering*’ (PCF 7). According to a PCF and a manager from the same service, commonalities with the PCF regarding a migrant experience also seemed to facilitate parents’ engagement. ‘*There’s some sort of connection of experience*’ (PCF, number omitted).

Creating a relationship with parents could also involve including all primary caregivers of the children, as mentioned by a PCF. ‘*Both* [parents] *are getting the information, and we try to keep including both in everything*’ (PCF 4).

#### 3.2.2 Tailoring support

Based on PCFs’ and managers’ views, tailoring support according to families’ needs and characteristics/circumstances seemed relevant to improving parents’ engagement and the potential benefits of the intervention. ‘*The main thing is how to do it* [support] *in an efficient way. One will be to create strong relationships. Another thing is to kind of adjust the content to what’s really needed’* (PCF 2).

PCFs and managers acknowledged the importance of including parents’ views in planning. *‘Involving parents in creating the events, asking them before, so they are kind of co-creating it’* (PCF 2). It could encompass asking parents about their needs and interests. *‘The PCF asks: Do you have any interest? Would you like to engage in the courses? Is there anything I can do for you at all?’* (Manager 6). Considering families’ views in planning future activities could also involve inviting children and parents to share their thoughts about past activities. ‘*I check with the children and parents and say: How did that go? What did you think if we were to do it again? What might we change?’* (PCF 8).

Related to tailoring support, a PCF and a manager highlighted the importance of reflecting on the implementation of parenting support courses, such as Parents Plus within Powerful Parenting. It could include selecting only some topics according to families’ needs and strengths. *‘Parents Plus covers the whole range of parenting. When we do assessments, we realise this parent is really good with routine or whether the parent’s struggle is with managing behaviour. So we only work with the parents on that aspect’* (Manager 1).

#### 3.2.3 Adaptation of activities during the pandemic

Due to COVID-19-related social distancing measures, PCFs and managers adapted the delivery of activities, such as remote communication and meeting outdoors, to continue supporting parents. *‘We try to think of different ways that we can keep that engagement process going’* (PCF 4).

### 3.3 Theme 3. Implementation drivers

The drivers of Powerful Parenting implementation seemed to include the PCF role’s requirements, training and support opportunities for PCFs, the collaboration between the PCFs, the ECEC service and other services, and funding.

#### 3.3.1 PCF role’s requirements

PCFs work with parents who might engage differently and whose families might have diverse needs. Hence, based on PCFs’ and managers’ views, the PCF role could require practitioners with particular characteristics. *‘Somebody that has childcare experience, and maybe mental health experience, or some level of emotional intelligence to be able to support families where they are at’* (PCF 3). Being able to accompany parents’ pace could entail flexibility from the PCFs. *‘We’re constantly reinventing the wheel’* (PCF 5).

#### 3.3.2 Training and support opportunities for PCFs

Considering PCFs’ and managers’ reports, training, sharing and networking opportunities were potentiated through the CoPs, which seemed to support the work with parents. *‘The COPs can be a good learning point because we can suggest what we’d like. For instance, getting someone talking about grief. It’s good to have their support* [the other PCFs] *like a network as well’* (PCF 4). Guidelines and monitoring of the work developed also seemed a valued aspect. ‘*It has improved a lot the quality of the supports we are doing*’ (PCF 7).

However, PCFs also suggested considering more opportunities for exchanging support among them, such as visiting other ECEC services, pairing a new PCF with another with more experience during induction, and meeting outside the COPs. *‘I would suggest, maybe, informal meetings. We have the COPs, and the PCFs would meet, like, once every month or two months, to kind of support each other’* (PCF 6). The online group created with PCFs was also acknowledged as helpful because ‘*everyone’s able to interact with each other and share information*’ (PFC 6).

#### 3.3.3 Collaboration with the ECEC service and other services

Based on PCFs’ and managers’ reports, the PCFs could meet with the managers and ECEC staff to plan activities collaboratively when needs were identified. ‘*It [planning] would come from talking to the parents. Then, input from the childcare service itself and the manager*’ (PCF 3). The PCF could also work with parents to complement the ECEC practitioners’ work with the children. ‘*If the staff are working on something around child development with the child and, then, with the parent, the PCF may be able to do an extra piece, maybe looking into something that would support the parent in that*’ (Manager 5).

Collaboration with other services (besides the ECEC service) could occur to refer families and implement support/activities (e.g., the delivery of food packs). A PCF suggested creating ongoing collaborations with services: *‘Maybe trying to find partners to support different areas’* (PCF 7).

#### 3.3.4 Funding

PCFs described the financial support allocated to the ECEC services as helpful. *‘We have budgets there to provide for families and provide for different activities, which takes huge pressure off the service itself’* (PCF 4). However, according to both PCFs and managers, increasing the PCFs’ funded working hours to more than 25 a week would enable supporting more parents, particularly those whose availability does not match the PCF’s working schedule. *‘It’s a 25-h-a-week programme for services that run from nine to half-five. We do not have the PCF every day in the afternoon, and some parents are missing out’* (Manager 6).

A PCF and a manager suggested that it could be important to allocate funding per service based on the number of families being supported (rather than the same amount for all services). A manager also indicated concerns regarding the funding being provided annually. ‘*It’s on my mind every year: will the funding change, will it go down, would it be any problems? Because that’s not good for staff to work on a year-to-year basis’* (Manager 1).

### 3.4 Theme 4. Relevance of the model

The availability of a role dedicated to supporting parents in the ECEC service, a focus on multiple needs, and perceived benefits suggested the relevance of a parenting support model as Powerful Parenting.

#### 3.4.1 A role dedicated to supporting parents

By having *‘the time and the space’* (PCF 3), PCFs seemed to be able to provide further support for parents beyond that from the ECEC staff working directly with children, according to PCFs and managers. ‘*The supports that we are providing are just impossible to get in other places when there is not a person just specifically for parental engagement*’ (PCF 7). *‘It is someone there that has the time to spend with the parent, is not rushing back out to the class, will do follow-ups, look for supports, do calls and make the link’* (Manager 4).

PCFs and managers noted that the availability of support through the PCF role seemed particularly relevant during the pandemic. ‘*When the lockdown came, the PCF would go outdoors, and was able to truthfully speak to the parents about the children because she knew the children. We felt that the engagement with the parents through that was really taken off’* (Manager 6).

#### 3.4.2 A focus on multiple needs

PCFs indicated working with parents with diverse needs. These could be related to parenting and children’s development, socio-emotional wellbeing and difficult life situations (e.g., financial hardship). *‘They want practical help and support with their children’s developmental delay, or housing crisis, their addiction, that kind of stuff’* (PCF 5). Managers seemed to acknowledge the focus on diverse needs as a positive aspect of the model. *‘It’s a great benefit for parents to have, for whatever matter or issue parents might have or need, whether it could be around children or themselves’* (Manager 2).

#### 3.4.3 Perceived benefits

Although the success of the support could vary, PCFs and managers perceived benefits for parents. *‘Parents would give me positive feedback about the impact that having the support has on their life, their children’s life. Maybe it’s parenting support, or they did the Parents Plus Programme, and they can see such a change in their world and in their child’s behaviour’* (PCF 6). The benefits could include ‘*access to more supports and referrals to relevant services meeting the parents’ needs’* (Manager 3).

Based on managers’ views, the PCF role seemed to reinforce the link between the service and parents. *‘There is a connection between the families and the service, a different layer’* (Manager 3). Considering the activities organised with the ECEC service, PCFs seemed to perceive a positive effect on the connection between families. ‘*It helps them to build like a sense of identity and belonging. They’re part of like a group*’ (PCF 8). One PCF also mentioned potential benefits for the ECEC staff. ‘*I think everyone, even the staff, gets involved and gets some learning from what we are doing*’ (PCF 7).

As a way of exploring perceived outcomes associated with the support offered, as part of the research question guiding this study, PCFs were also asked to score the extent to which they perceived that Powerful Parenting was addressing/strengthening a set of outcomes. These outcomes correspond to those that Powerful Parenting aims to achieve based on the formulation of the model. Table 2 shows the means and medians obtained. Overall, the results suggested that they viewed the model as contributing to positive outcomes regarding parenting, parents-ECEC service relationship, and involvement of families in community services. The Home Learning Environment had the lowest score, which could be related to fewer opportunities to offer support based on observed practices, ‘*especially this year, not being able to go there* [the family’s home]’ (PCF 2). Another PCF indicated that activities related to the Home Learning Environment could not be a priority for some families experiencing difficult life situations. ‘*Some practical help with budgeting, food, and case conferences with child protection, that kind of stuff, is kind of more of their priority’* (PCF 5).

**Table 2:** PCFs’ perceived outcomes of Powerful Parenting (from one = low to five = high).

Perceived outcomes	n	Mean(SD)	Median
Strengthen parenting skills	8	4.13(0.64)	4
Help parents/carers to understand the child’s development	5	4.20(0.45)	4
Provide parents/carers with new ways to interact with children	7	3.86(0.69)	4
Promote the home learning environment	8	3.63(0.92)	3
Help families prepare for a smoother transition of children to school	7	4.71(0.48)	5
Promote improved partnerships between the parents/carers and the ECEC services	7	4.57(0.53)	5
Promote the engagement of both parents of the child or other carers in children’s education	8	4.50(0.53)	4.5
Involve families with other community services	7	4.14(0.69)	4

## 4 Discussion

This study aimed to explore factors potentially affecting parents’ engagement in Powerful Parenting and associated outcomes by exploring the views of PCFs and ECEC managers involved in delivering it. Studying this model can contribute to informing about factors affecting engagement and outcomes regarding parenting support in ECEC services.

Overall, there seemed to be a high engagement of the parents with the activities/support offered. Within a study with 27 parents who shared their views on Powerful Parenting, most indicated a high motivation to talk with or participate in activities organised by the PCF (Leitão and Shumba, 2024). However, according to PCFs and managers, the engagement could vary depending on the parents’ circumstances and type of activity. Simultaneous commitments (work, minding children), lack of interest or confidence, different language, difficult life situations, and perceiving the model as similar to other services could be barriers to engagement, as identified in previous studies (de Greef et al., 2018; Koerting et al., 2013). Regarding the type of activity, parents seemed to look for one-to-one meetings, which could enable tailored support, and activities with children or whose rationale was for children’s benefit.

Identified approaches to promoting engagement and responsiveness to families’ needs included building a trusting relationship with parents by respecting their pace, using an empathic/non-judgemental approach and sharing the support’s rationale, as well as tailoring support according to families’ needs. Parents were also found to value PCFs’ being approachable and responsive to their needs (Leitão and Shumba, 2024).

A trusting relationship and open communication with parents, supporting them in decisions, and a focus on the child’s wellbeing can foster parents’ engagement in the support offered and positive outcomes for children’s development and families’ wellbeing (Cohen et al., 2020; Cook et al., 2023; dos Santos et al., 2024; Jiménez et al., 2024). Although service flexibility might vary in degree and nature (Cadima et al., 2017), tailored support can also promote parents’ engagement (Anders et al., 2019; Devaney et al., 2021). Attending to parents’ views and perceiving parents as experts in their lives, aspects identified in this study, can be part of a strengths-based approach (Canavan et al., 2016; Dunst et al., 2007). Of note, while parents were acknowledged as experts in their lives, a barrier to engagement identified in this study referred to parents being unaware of their expertise in their children’s education, highlighting the importance of dialogue with parents/families towards a shared vision.

Regarding drivers of the model implementation, the PCF role seemed to require the competences to be flexible to move at the parents’ pace and establish trusting relationships. These competences have been recognised as relevant to promoting parents’ engagement and implementation quality of parenting/family supports (Cohen et al., 2020; dos Santos et al., 2024; Leitão et al., 2023). Although sharing a migrant experience with parents was not a criterion in the PCFs’ recruitment (such as when involving target group members as practitioners), it was perceived as facilitating engagement in one ECEC service. Future research could be relevant to explore the role and mechanisms of a shared migrant experience (Cohen et al., 2020).

Training, sharing among PCFs and with the other ECEC practitioners, and collaboration opportunities were also identified as implementation drivers. Learning from other practitioners working with families, which CoPs can potentiate, has been identified as an effective way of developing professionally (Slot et al., 2018). Parenting support is more likely to be effective when practitioners receive adequate and ongoing training and support (Bernedo et al., 2024; WHO, 2022). Collaboration with other services has also been recognised as enabling the implementation of family/parenting support (Cadima et al., 2017).

The relevance of a parenting support model such as Powerful Parenting encompassed having an available person dedicated to working with parents in ECEC services, including on more than one area of need, which can be key to effective parenting support (Cadima et al., 2017; Dunst and Trivette, 2009). Even though the success of the support could vary, PCFs and managers perceived benefits from the model to parents in terms parenting, access to other services or resources, and parents-ECEC service relationship. Based on parents’ views, the PCF role appears as a central point of contact, bridging the home and the classroom/service, which could be of benefit to children, parents and other ECEC practitioners; the perceived benefits included increased understanding of children’s needs and how to address them, facilitated contact with services, and socio-emotional benefits, such as reduced stress (Leitão and Shumba, 2024). Enhancing the connection between the parents and ECEC services can reinforce the latter as communities of care and a sense of belonging, as found in Ireland (Garrity and Canavan, 2017). In turn, this connection can support children’s development (Barnett et al., 2020; Sim et al., 2021).

In the current study, no specific children’s outcomes were identified. Although parent wellbeing can enhance parenting practices and child outcomes (Dunst, 2022), evaluating Powerful Parenting’s impacts, for instance, based on a model’s theory of change and considering the dosage received, could allow exploring potential effects on parents and children.

Regarding the results of this study, the authors recognise common points between the different themes and sub-themes (e.g., barriers to engagement could be related to approaches to promote engagement and responsiveness to needs). Nonetheless, the authors (coders) sought to generate the themes and subthemes based on the data using an inductive approach, discussing them while revisiting the data.

The authors also recognise that while the COVID-19 pandemic, a frequent topic in this study, is not as challenging as it used to be at the time of the research, some factors affecting engagement in Powerful Parenting and associated outcomes may continue to be applicable, and inform future parenting support provision. However, researching Powerful Parenting in a post-pandemic context can provide relevant information on factors affecting engagements and potential outcomes.

### 4.1 Limitations

The researcher conducting the interviews was recruited by the organisation coordinating Powerful Parenting to evaluate this model. This might have posed challenges for the participants to share their critical views openly, even if informed that personal/service identifiers would be omitted from the results provided to the organisation. Different methods to collect and analyse the data could be relevant to address potential bias, such as observation (not possible at the time of the study due to social distance measures). A multi-informant approach that included the views of children, parents, and other staff would allow a more comprehensive understanding of Powerful Parenting. Parents, children, and professionals can be actors in the construction processes of parenting, and their perspectives should be considered when evaluating support effectiveness (Baird and Grace, 2017; Geens and Vandenbroeck, 2013).

Data were not analysed per service to avoid the identification of participants. Collecting data on the dosage/attendance per activity or service was not possible. However, variation in the implemented activities/supports across ECEC services could be expected when parents’/families’ needs are considered. Additionally, both engagement and perceived outcomes could be associated with PCFs’ characteristics (e.g., professional experience) and/or with ECEC services’ aspects. Future research could focus on links between particular activities/supports, engagement and outcomes to explore which activities work best and for whom.

### 4.2 Implications for policy and practice

The reported overall engagement of parents and perceived benefits suggested that parenting support within ECEC services through a dedicated role, such as the PCF, can be relevant for parents. Creating a specialist role in ECEC services to support the work with parents is a proposed measure to potentiate parental engagement (European Commission, 2021). Building parenting supports on existing delivery platforms can foster their scaling up and sustainability (Britto et al., 2017; Skattebol et al., 2023). Additionally, easy access to support can lower the potential stigma associated with seeking/receiving assistance and increase its use (Molinuevo, 2013; Smith, 2019).

In Ireland, where 94% of children from 3 to 6 years old were in formal childcare/education in 2023 (Eurostat, 2025), national policy recognises the importance of partnerships with parents in ECEC (CECDE, 2006; NCCA, 2009), and ECEC services as potential delivery mechanisms of support for parents/families (DCEDIY, 2024; DCYA, 2018). However, in the national context, parents and practitioners working with families reported needs related to enhancing access to parenting support (DCEDIY, 2020, 2021; Hickey and Leckey, 2021), and there were identified challenges securing sustainable family support advancements (Churchill et al., 2020). A model such as Powerful Parenting can contribute to informing the development of accessible neds-led supports, a goal of the current national model of parenting support services (DCEDIY, 2022). It can also inform the recent government commitments to enable the role of ECEC services in signposting parents to existing parenting supports and/or providing them in the settings (DCEDIY, 2024).

Based on the findings of this study, some factors were identified as relevant to promoting parents’ engagement in parenting support and associated benefits. Available support during a broader schedule (e.g., more than 25 h a week), which requires appropriate funding, can facilitate access to support for parents with work or other commitments. Support to face language barriers and promoting practitioners’ intercultural competences can enhance the engagement of diverse parents (Slot and Nata, 2019). Recruiting staff with good interpersonal skills seems relevant to building trusting relationships with parents (Anders et al., 2019; Cohen et al., 2020), which can be supported by high-quality training, opportunities for peer-to-peer learning, and organisational support. Finally, including parents in planning and decision-making and considering the availability of one-to-one and group activities can potentiate supports that respond to their needs and resources (Britto et al., 2017; Connolly and Devaney, 2018; Jiménez et al., 2024).

## Data Availability

The datasets presented in this article are not readily available because of concerns regarding participant anonymity. Requests to access the datasets should be directed to catarinafcl@gmail.com.

## Footnotes

^1^ www.parentsplus.ie

^2^ www.peeple.org.uk

^3^ www.incredibleyears.com
