# Supplementary material for: Parenting support in ECEC services: the views of practitioners implementing a model in the Irish context about parents’ engagement and associated outcomes
Source: Front Sociol. 2025 Apr 25;10:1489477. doi: 10.3389/fsoc.2025.1489477 (PMC12061993; doi:10.3389/fsoc.2025.1489477)
Supplement: Supplementary file 1 [file Table_1.docx]

Supplementary Material

Table ST1. Number of referrals to other services during the academic year reported by PCFs.

| **Type of service** | **n** |
| --- | --- |
| Financial support (e.g., social welfare, childcare costs) | 25 |
| Assessment of the child’s health needs | 23 |
| Access and inclusion supports focused on children’s needs | 41 |
| Child protection | 10 |
| Speech and language development | 32 |
| Early intervention to support children with unmet additional or complex needs | 4 |

Note. The number of referrals shown corresponds to the quarterly sum and can be related to the same family.

Table ST2. Examples of supports/activities implemented within Powerful Parenting referred by PCFs and managers.

| **Delivery mode** | **Type of support** | **Examples** |
| --- | --- | --- |
| One-to-one support | Identification of needs | Checking how the families are (e.g., during morning drop-offs) |
|  | Informational support | Sharing of information on parenting (e.g., on children’s transition to school; ideas for activities with children)  Sharing of information on job opportunities for parents |
|  | Emotional support | Listening to the parents |
|  | Practical support | Help with accessing services/support for children with additional needs (e.g., speech and language)  Help with accessing services/support for families’ difficult life circumstances or experiences (e.g. addictions, poverty)  Links with dental services |
|  | Instrumental support | Delivery of resources at families’ homes (e.g., packs to do family activities at home, food packs, materials for preparing for the transition to school) |
| Group activities with parents | Informational support | Coffee mornings and online meetings with parents (e.g., mental health, transition to school, and speech and language therapy)  Parents Plus Programme |
| Group activities with parents and children | | Family mornings: Online (e.g., dance and fitness, baking, bedtime stories, play therapy); In the ECEC service (e.g., gardening, painting, calendar events such as Christmas, International Father’s Day)  Summer activities (e.g., on the transition to the next academic year) |

Note. The examples were identified using a deductive approach, in which the delivery modes and types of support were predefined categories following the activities that could be offered within Powerful Parenting.
